# Characterization of Rheumatoid Arthritis Subtypes Using Symptom Profiles, Clinical Chemistry and Metabolomics Measurements

**DOI:** 10.1371/journal.pone.0044331

**Published:** 2012-09-12

**Authors:** Herman A. van Wietmarschen, Weidong Dai, Anita J. van der Kooij, Theo H. Reijmers, Yan Schroën, Mei Wang, Zhiliang Xu, Xinchang Wang, Hongwei Kong, Guowang Xu, Thomas Hankemeier, Jacqueline J. Meulman, Jan van der Greef

**Affiliations:** 1 Division of Analytical Biosciences, LACDR, Leiden University, Leiden, The Netherlands; 2 Sino-Dutch centre for Preventive and Personalized Medicine, Zeist, The Netherlands; 3 CAS Key Laboratory of Separation Science for Analytical Chemistry, Dalian Institute of Chemical Physics, Chinese Academy of Sciences, Dalian, China; 4 Mathematical Institute, Leiden University, Leiden, The Netherlands; 5 Netherlands Metabolomics Centre, Leiden University, Leiden, The Netherlands; 6 Oxrider, Education and Research, Nieuwegein, The Netherlands; 7 SU BioMedicine, Utrechtseweg, Zeist, The Netherlands; 8 College of Life Science Company Limited, Hangzhou, China; 9 Zhejiang Xinhua hospital Rheumatology and Immunology Department, HangZhou, China; 10 TNO Netherlands Organization for Applied Scientific Research, TNO Pharma, Zeist, The Netherlands; University of Michigan, United States of America

## Abstract

**Objective:**

The aim is to characterize subgroups or phenotypes of rheumatoid arthritis (RA) patients using a systems biology approach. The discovery of subtypes of rheumatoid arthritis patients is an essential research area for the improvement of response to therapy and the development of personalized medicine strategies.

**Methods:**

In this study, 39 RA patients are phenotyped using clinical chemistry measurements, urine and plasma metabolomics analysis and symptom profiles. In addition, a Chinese medicine expert classified each RA patient as a Cold or Heat type according to Chinese medicine theory. Multivariate data analysis techniques are employed to detect and validate biochemical and symptom relationships with the classification.

**Results:**

The questionnaire items ‘Red joints’, ‘Swollen joints’, ‘Warm joints’ suggest differences in the level of inflammation between the groups although c-reactive protein (CRP) and rheumatoid factor (RHF) levels were equal. Multivariate analysis of the urine metabolomics data revealed that the levels of 11 acylcarnitines were lower in the Cold RA than in the Heat RA patients, suggesting differences in muscle breakdown. Additionally, higher dehydroepiandrosterone sulfate (DHEAS) levels in Heat patients compared to Cold patients were found suggesting that the Cold RA group has a more suppressed hypothalamic-pituitary-adrenal (HPA) axis function.

**Conclusion:**

Significant and relevant biochemical differences are found between Cold and Heat RA patients. Differences in immune function, HPA axis involvement and muscle breakdown point towards opportunities to tailor disease management strategies to each of the subgroups RA patient.

## Introduction

Discovering subtypes of rheumatoid arthritis (RA) patients is considered a key research area for the improvement of response to therapy [1], [2]. RA is a heterogeneous disease which is illustrated by the very good response of some patients to a biological therapy, but a complete lack of response in a large number of other patients [3]. Another striking observation is that in a large group of RA patients low disease activity or remission can be achieved using a single conventional disease-modifying anti-rheumatic drug (DMARD), which contrasts with the current viewpoint to offer aggressive therapy in an early stage of the disease to all patients [4]. Personalized medicine aims to provide the information that allows targeting the right treatment option to the right patient [5]. The first step in this approach is to find relevant subtypes of patients for which a different treatment strategy would clearly be beneficial.

Several subtypes of RA patients have been identified based on particular clinical and molecular features [6], [7]. Markers such as disease duration and age have been identified that predict response to treatment [8], [9]. Although some molecular markers have been found to predict functional and structural outcomes, these markers rarely find their way into clinical practice. One reason is the difficulty to translate markers found in trial populations to routinely measurable and cost-effective predictors for individuals [10]. This indicates that there is a need to develop new robust and reliable clinically applicable tools to identify subtypes of patients.

Discovery of novel relevant subtypes of RA patients could be improved by using prior knowledge. In this study a Chinese perspective on subtypes of RA patients is used to focus the analysis of the data. According to this perspective RA patients can be divided in two groups (Cold RA and Heat RA) which are treated very differently in Chinese medical practice [11], [12].

Cold and Heat are general concepts used in Chinese medicine to distinguish between two types of reactions of the body to some disturbance [13]. A Cold reaction is characterized by pallor, intolerance of cold, absence of thirst, loose stools, clear profuse urine, a pale tongue and a slow pulse. A Heat reaction is characterized by flushed face, fever, thirst, irritability, restlessness, constipation, deep-colored urine, reddened tongue and a rapid pulse [14]. These two types of reactions are expressed in any type of disease to a certain extend. However, Cold and Heat are especially important for rheumatoid arthritis because this disease is perceived in classical Chinese medicine as the result of an invasion of three out of the four existing external pathogens: Wind, Cold, Heat and Damp [13].

Some work has been done to elucidate biological mechanisms related to Cold and Heat types of RA patients. In 2009 we measured 64 differently expressed genes in CD4 positive T-cells of RA patients. This set of genes was enriched for the immune system functions and especially for apoptosis regulation. In Heat RA patients apoptosis related genes were upregulated while in Cold patients apoptosis resitance genes were upregulated [11]. Additionally, a number of plasma metabolite concentrations was significantly different between Cold and Heat RA. Later, genes related to calcium signaling, cell adhesion, PPAR signaling and fatty acid metabolism were found in CD4 positive T-cells of Heat RA patients [15]. Toll-like receptor signaling related genes were found in the T-cells of Cold RA patients. In a similar study, Cold RA was related to Alanine, aspartate and tyosine metabolism and Heat RA to the MAPK pathway, Wnt signaling and insulin signaling, also found by measuring gene expression in CD4 positive T-cells [16]. A GC-MS analysis of Cold and Heat RA plasma revealed elevated plasma levels of glycochenodeoxycholate, proline, saturated and mono-unsaturated phosphatidylcholine (PC) but decreased levels of urea, free fatty acid (FFA) and polyunsaturated PC in Heat RA compared to Cold RA [17].

Recently it was shown that Cold RA patients respond much better to a combination therapy with diclofenac, methotrexate and sulfasalazine than Heat RA patients [18]. However, in clinical studies and clinical practice the features of Cold and Heat might be valued differently by each Chinese medicine practitioner. To improve acceptance of this classification in Western clinical practice it is important to standardize the classification. Further characterization of the physiological, constitutional and biological differences between the Cold and Heat subtypes of RA patients is valuable for increasing our understanding of the disease mechanism.

In this study a systems biology strategy is employed to study the differences between Cold and Heat types of RA patients, classified by a Chinese medicine expert. Data were collected on symptoms, clinical blood parameters and urine metabolites. Multivariate statistics were employed to discover the most discriminating features for the two groups. In particular, a categorical principal component analysis [19], [20] was used for the questionnaire and clinical chemistry data and partial least squares discriminant analysis is used for the metabolomics data [21]. The questionnaire was used to determine symptom patterns related to Cold and Heat RA that could be used in clinical practice to determine these subtypes in an objective manner. The metabolomics and clinical results were related to biological processes and related to current understanding of the heterogeneity of rheumatoid arthritis.

## Subjects and Methods

### Subjects

Female subjects were recruited in the Zhejiang Xinhua Hospital in 2010. The institutional review board of the hospital gave written approval and judged that the study was conducted according to the ethical guidelines. A doctor explained the details of the study to the subjects and ensured written informed consent of each subject. All eligible subjects were diagnosed by a rheumatologist as rheumatoid arthritis patients according to the ACR criteria [22]. Inclusion criteria were a female gender due to the much higher prevalence of RA in women and age >18 years. Each patient was classified by the same Chinese medicine expert as either a Heat subtype or a Cold subtype. When the subtype was unclear the patient was not included in the study. General information such as age, disease duration, medication, etc. was collected using a standard form. Student’s t-Test (two-tailed) was used to evaluate the differences between the two groups. Furthermore, a symptom questionnaire was completed by all the patients. Blood and urine samples were taken from each patient after fasting overnight for routine clinical measurements and metabolomics analysis.

### Symptom Questionnaire

A short version of a recently developed systems diagnosis questionnaire was used in this study [23]. The 106 item questionnaire was shortened to 57 items by two Chinese medicine experts to target it more directly to Cold and Heat related symptoms. The final version of the questionnaire contained items related to five categories of symptoms: breathing, digestion, climate, quality of the symptoms and pain. The questionnaire was translated into Chinese. An English language version of the questionnaire is added as supplementary information (Text S1). The consistency of the data was checked and the data were recoded [23].

### Clinical Measurements

Routine clinical chemistry measurements were conducted on fasting blood samples by the Zhejiang Xinhua Hospital. These measurements consisted of the complete blood count and the following serum measurements: uric acid (URIC), creatinine (CREA), blood urea nitrogen (BUN), triglycerides (TG), cholesterol (CHOL), total protein (TP), albumin (ALB), globulines (BIB), aspartate aminotransferase (AST), alanine aminotransferase (ALT), creatine kinase (CK), creatine kinase-MB (CKMB), lactate dehydrogenase (LDH), immunoglobulin A (IGA), Immunoglobulin G (IGG), immunoglobulin M (IGM), complement 3 (C3), complement 4 (C4), antistreptolysin O (ASO), rheumatoid factor (RHF), c-reactive protein (CRP), anti-cyclic citrullinated protein antibodies (CCP). The results of each clinical parameter were uniformly binned into 19 categories to increase the robustness [19].

### Metabolomics Measurements

Urine samples were prepared for Ultra Fast LC/MS-IT-TOF (Shimadzu, Japan) analysis [24]. The technical variation of the analysis over time was monitored by a pooled quality control (QC) sample strategy [25]. The analysis was performed on a Ultra Fast LC/MS-IT-TOF with gradient elution using 0.1% formic acid in water as mobile phase A and 0.1% formic acid in acetonitrile as mobile phase B. For the LC a T3 column (ACQUITY UPLC HSS T3 1.8um, 2.1×100 mm, Waters) was used. Ion mode was switched between positive and negative mode between each scan [24].

Sample preparation for the plasma metabolomics analysis was conducted as follows: 200 µL plasma was mixed with 800 µL acetonitrile, then the mixture was centrifuged at 14000 rpm (Biofuge Stratos, Thermo Scientific, USA) for 10 min at 4°C. The supernatant was freeze-dried and redissolved with 200 µL of 20% acetonitrile.

Chromatographic separation was performed on a BEH C8 column (10 cm×2.1 mm, 1.7 µm, Waters, USA) using a Thermo Fisher Accela LC system. The gradient duration was 31 min at a flow rate of 300 µL/min with mobile phase (A) 0.1% formic acid solution and (B) acetonitrile. The injection volume was 6 µL. The samples were analyzed randomly and the same QC strategy was used as in the urine analysis.

An LC-MS method was used to measure the plasma metabolites. The LTQ-Orbitrap XL (Thermo-Fisher Scientific) settings were as follows: capillary temperature 325°C, source voltage 4.5 kV, capillary voltage 49 V for ESI+analysis; capillary temperature 325°C, source voltage −3.5 kV, capillary voltage −40 V for ESI- analysis. The mass range was set at 100–1000 m/z. The resolution of the Orbitrap was set at 30000.

The urine metabolomics measurements resulted in a list of features after preprocessing the data using Profiling Solution (Shimadzu, Japan) [24]. The plasma metabolomics data were processed by SIEVE software (V1.2, Thermo-Fisher Scientific) for peak picking and peak alignment. The parameter of MZ width was set at 0.01 Da and RT width was set at 0.6 min. Gradient peaks were removed. Features not present in at least 80% of the samples in one class were removed. Zeros were replaced by the smallest measured value divided by 2, the urine data were mean centered and the plasma data was autoscaled. Principal component analysis was then used to screen the data for outliers (objects with scores deviating from the scores of the bulk of the samples) [26], to determine the stability of the analysisover time (by comparing the scores of the QC samples) and monitor possible batch effects (by checking possible time trends in the scores of the objects).

### Statistical Analysis

#### Questionnaire and clinical chemistry data

The focus of the data analysis is to identify questionnaire items and clinical parameters that distinguish between the subgroups of RA patients classified by the CM expert. A forced classification approach [27] using Nonlinear Principal Component Analysis (NLPCA) was chosen. NLPCA is a Principal Component Analysis method that is suitable for variables with mixed measurement level and variables that may have nonlinear relationships to each other [19]. The possibility to capture nonlinear relationships is important because the human body is a nonlinear system expressing complex behavior [28]. Such complex behavior has been observed in the immune system that plays a key role in rheumatoid arthritis. For instance cytokine dose response curves show an initial threshold and stable attractors have been found in cytokine systems [29]. More important is the ability of NLPCA to deal with the questionnaire data that are measured on an ordinal scale or as binary data. The questionnaire data analysis approach using NLPCA is described in detail in a previous paper [23].

NLPCA uses an ‘optimal scaling’ approach to find quantifications for categorical variables and optimal transformations for interval variables, which means that the percentage of variance of the transformed variables accounted for by the principal components is maximal [30]. The questionnaire items and clinical parameters were all included in a single NLPCA analysis allowing the interpretation of relationships between questionnaire items and clinical parameters.

The use of a the classification variable with a very large weight, here 1000 will cause the objects to cluster together into subclouds in the object space. The weight was chosen such that the objects of both classes were separated completely. As the classification variable is a binary variable, one principal component is sufficient to describe the class differences. In this study we used CATPCA in SPSS version 17.0 [31].

An important consideration in NLPCA is the analysis level chosen for the variables [20]. For the questionnaire variables the analysis level was kept the same as the measurement level (39 ordinal and 3 nominal). Subsequently the presence of nonlinear relationships between the clinical chemistry variables and the classification variable was examined by comparing numerical, spline ordinal and spline nominal transformations of the clinical chemistry variables (i.e., from most restricted to least restricted transformations). A substantial increase in the total variance accounted for (total VAF) due to an analysis level with more degrees of freedom then verifies that a less restrictive model is more appropriate.

After choosing the appropriate analysis level, variables with a proportion variance accounted for (VAF) >0.20 were selected for further analysis to increase the stability of the model and to focus on the most important variables. Leave two out cross validation was used to estimate the classification error of the model. In this approach two objects are reserved as a validation set while a NLPCA model is built using the other 37 objects. The class of the two left out objects is then predicted using the NLPCA model. This procedure is repeated until each of the objects is left out once and the class of each object is predicted. An overall classification error is subsequently calculated and a final model is built using all the available objects. Permutation testing was used to check whether this final model is different from a model based on a random classification.

#### Metabolomics data

A partial least squares discriminant analysis (PLS-DA) model was built for both the urine and plasma data set to find the most discriminating features between the Cold RA and Heat RA groups, a standard, well accepted technique used in the metabolomics field to discover characteristic differences between groups [32]. A separate model for the urine and plasma data was built to examine the performance of a metabolite profile measured in one compartment in a single analysis. Such a profile would be much easier to develop into a diagnostic tool that can be applied in a clinical setting than a combination of two metabolomics profiles.

A tenfold double cross-validation scheme was followed by permutation testing (250 times) [33]. The inner loop of the cross-validation scheme is used to estimate the optimal number of principal components. In this inner loop two objects are left out to validate the number of principal components. The outer loop is used to estimate the error rate of the model. Again, in the outer loop two objects are left out to independently estimate the error rate. Variables were selected using a jack-knifing procedure in which variables with a standard error in the regression vector above a threshold were removed from the dataset. This threshold is decreased stepwise until the error rate of the model starts to increase. The model with the lowest error rate is then chosen as the optimal model and a final model is then built for the corresponding set of variables and including all the objects. Subsequently, the significance of the resulting model is determined by permutation testing. The same double cross-validation procedure is used to build a model of the same data set but with a permuted classification. After 250 times permuting the classification, the classification error of the 250 models resulting models is compared with the classification error of the model based on the correct classification. The error rate of the correct classification model should be lower than 95% of the distribution of error rates resulting from the permuted classifications.

Identification of the most discriminating features indicated by the PLS-DA model was then attempted for both the urine and plasma model [34]. Evidence for the identity of features was accumulated by integrating information on the following: accurate mass of the ions and some fragments, retention time, HMDB database and in house human urine compound lists. The identification of some compounds was then verified by authentic standards. Finally, a biological interpretation was sought for the most discriminating features.

## Results

### Subjects

In total 50 patients were enrolled in the study. Eleven of these patients did not fulfill the inclusion criteria and were removed from the analysis. Seven of those eleven patients were male and therefore excluded, of two persons there was no RA diagnosis available, one patient was in the hospital for other reasons and therefore excluded and finally one person was excluded from this analysis because the symptom questionnaire was not completed. Finally, 20 RA patients of the Cold subtype and 19 of the Heat subtype were retained in the study.

Table 1 shows the patient characteristics per group expressed as the means of the age, disease duration, height and weight. None of these characteristics were significantly different (p-value <0.05) between the Cold RA and Heat RA patient groups. 34 patients received western medication such as methotrexate, sulfasalazine. other DMARD’s and NSAID’s. 36 patients received Chinese herbal medicine.

**Table 1:** Patient characteristics.

	Cold (n = 20)	Heat (n = 19)	p
Age (years)	51±13 (24–74)	54±11 (34–77)	0.44
Disease duration (months)	90±90 (4–240)	100±100 (24–348)	0.44
Height (cm)	159±4 (150–167)	160±4 (155–170)	0.50
Weight (kg)	58±18 (40–120)	58±9 (37–75)	0.97

* Range is given in parenthesis.

### Clinical and Symptom Differences between RA Subtypes

After merging scoring categories containing less than 7 observations, 15 variables were left with only a single category. There is no variation between the objects for these variables which were therefore removed from the analysis: ‘Sudden shortness of breath’, ‘Amount of phlegm’, ‘Sticky phlegm’, ‘Colored phlegm’, ‘Tender lower abdomen’, ‘Aggravated with pressure’, ‘Smelly diarrhea’, ‘Chills S’, ‘Chills F’, ‘Fever with Chills’, ‘Pain aggravates at night’, ‘Stabbing pain’, ‘Sharp pain’, ‘Deep pain’, ‘Heavy pain’. After recoding, the data set used for further analysis contained 42 variables.

In the forced classification model the use of a 2^nd^ degree (quadratic) nonmonotonic spline (nominal analysis level) with 2 interior knots for the clinical measurements shows substantially larger variance accounted for (11.8%) than a monotonic spline (ordinal analysis level) (8.2%), indicating a nonmonotonic relationship between the measurements. Also, a two interior knot spline shows a larger variance (11.8%) than a one interior knot spline (9.8%) (more knots allow for more freedom in the transformation). Therefore a 2^nd^ degree spline nominal analysis level with two interior knots was chosen for the clinical chemistry variables. A new model was then built using this analysis level and used to select variables with a proportion of VAF >0.20. A model was subsequently built including the 18 selected variables. The model using splines with 2 interior knots for the clinical chemistry variables was then compared once more with a model using one interior knot. The results showed that the increase in total VAF in the model with 2 interior knots was mostly due to the variable MCHC. Therefore the final analysis level of the clinical variables was adjusted to a 2^nd^ degree spline with 1 interior knot, except for MCHC which was set to 2 interior knots. The final model had a total VAF of approximately 21% (see Table 2 for the loadings of the 18 variables). The transformation plots of the clinical chemistry variables of this model are included as supplementary information (Figure S1). Leave two out cross validation resulted in a classification error of 15%, which is substantially lower than the expected error rate based on random classification (which is 50%).

**Table 2:** Discriminating symptoms and clinical measurements.

Variable name	Loading
b17 MCHC	0.580
v40 Red joints	0.544
v39 Swollen joints	0.540
b37 IGG	0.504
v25 Warm feeling	0.495
b8 LY#	0.490
v41 Warm joints	0.490
v56 Dull pain	0.438
v50 Pain worsens by warmth and movement	0.399
b27 CHOL	0.370
b38 IGM	0.359
b19 PLT	0.285
b25 BUN	−0.374
b32 ALT	−0.419
v19 Cold feeling	−0.457
v29 Aversion to cold	−0.472
b18 RDW	−0.475
b44 CCP	−0.495

RA patients that are classified as Heat by the Chinese medicine expert are characterized with higher optimal quantifications on average on the symptoms ‘red joints’, ‘swollen joints’, ‘warm feeling’, ‘warm joints’, ‘dull pain’ and ‘pain that worsens with warmth and movement’, and with higher levels of the optimally scaled clinical parameters mean corpuscular hemoglobin concentration (MCHC), IgG, lymphocyte number (LY#), IgM, platelet count (PLT) and cholesterol. Cold RA patients are best characterized with higher optimal quantifications on average on the symptoms ‘cold feeling’ and ‘aversion to cold’, and higher levels of the optimally scaled clinical variables blood urea nitrogen (BUN), alanine aminotransferase (ALT), red blood cell distribution width (RDW) and anti-cyclic citrullinated protein antibodies (CCP).

Both groups of patients have a similar average score on the symptoms ‘pain’ and ‘stiff joints’ as well as on the clinical parameters rheumatoid factor and c-reactive protein.

### Urine and Plasma Metabolite Differences between RA Subtypes

Urine samples of 14 of the 19 Heat RA and 14 of the 20 Cold RA patients included in the analysis described above were collected successfully for the LC-MS analysis. In total 11 urine samples were either not collected at the hospital or the samples were not send to the laboratory for metabolomics analysis. PLS-DA analysis resulted in a final model containing 793 features. A classification error of 14% was obtained by a double cross-validation procedure. Permutation testing indicated that the error rate of this model is significantly (p<0.05) different from the distribution of models based on permuted classifications.

The tentative identification of the top 50 most discriminating metabolites resulted in three metabolites that were verified by comparing authentic standards with the samples: acetylcarnitine, riboflavin and pantothenic acid. Thirteen of the top features measured in positive ion mode as well as the acetylcarnitine standard showed the same neutral loss of mass 59.0747 by fragmentation, which is highly indicative of acylcarnitine compounds [35], [36]. The retention times of the features showing this loss was increasing with the mass, indicating higher mass features were less polar. This agrees with the fact that larger mass acylcarnitines have longer fatty acid chains and are therefore less polar. Furthermore, several glucuronides and soy isoflavones are suspected, but the identity was not further determined (Table 3).

**Table 3:** Top discriminating urine metabolites.

Importance	Compound	2Log Cold/Heat^c^
1	C8∶1 acylcarnitine^b^	−0.73
3	C10∶3 acylcarnitine^b^	−0.70
11	C8+OH acylcarnitine^b^	−0.89
13	acetylcarnitine^a^	−0.75
15	riboflavin^a^	−2.40
16	C11∶1 acylcarnitine^b^	−0.98
21	pantothenic acid^a^	−0.92
27	C6:DC acylcarnitine^b^	−0.55
31	C5 acylcarnitine^b^	−1.21
32	C10∶2 acylcarnitine^b^	−0.53
38	C8∶2 acylcarnitine^b^	−1.19
41	C10∶1 acylcarnitine^b^	−0.23
45	C9∶3 acylcarnitine^b^	−2.26
49	C10∶3 acylcarnitine isotope^b^	−0.66
50	C6∶2:DC acylcarnitine^b^	0.42

a verified with authentic standard.

b verified with characteristic 59 neutral loss, retention time order, accurate mass.

c Calculated 2Log of the ratio between average levels in Cold and Heat patients.

An LC-MS analysis of the plasma samples of 11 Cold RA and 14 Heat RA patients from the same set of patients that are used in the urine metabolomics and questionnaire analysis was performed. Three plasma samples were not available for analysis. Positive ion mode features were not included in the multivariate analysis because the QC samples were not stable. The PLS-DA analysis resulted in a model containing 214 features (classification error of 28%). Permutation testing indicated that the error rate of this model is significantly (p<0.05) different from the distribution of models based on permuted classifications. Some of the top 50 features resulting from the PLS-DA model could be identified with authentic standards (Table 4).

**Table 4:** Top discriminating plasma metabolites.

Importance	Compound^a^	2Log Cold/Heat^b^
2	Dehydroepiandrosterone sulfate (DHEA sulfate)	−0.96
4	4-Methyl-2-oxovaleric acid	−0.25
6	Indoxyl sulfate	−0.44
10	Uric acid	−0.25
11	Cholesterol sulfate	−0.65
13	3-Methyl-2-oxovaleric acid	−0.26
47	Tryptophan	−0.23
50	Alpha-ketoisovaleric acid	−0.13

a all compounds are verified with an authentic standard.

b Calculated Log of the ratio between average levels in Cold and Heat patients.

## Discussion

The subtypes of RA patients are characterized by four sets of features: clinical symptoms (questionnaire items), clinical chemistry measurements in blood, metabolite measurements in urine and in plasma. One hypothesis is that the Cold RA and Heat RA groups have a different inflammatory status. Of the most discriminating symptoms in the analysis, ‘warm joints’, ‘swollen joints’ and ‘red joints’ indicate a difference in inflammatory status. However, c-reactive protein (CRP), an important inflammation marker, was not retained in the final model because of a low proportion of total variance accounted for, based on the two groups. Rheumafactor (RF), an important marker for RA disease activity [37], was not retained in the model either. On the other hand, the lymphocyte numbers are higher in Heat RA patients, although this level is not considered elevated and indicative of inflammation in standard evaluation [38]. A comparable average disease duration of 90 and 100 months for the two groups suggests that not one of the groups can be considered an early arthritis or early aggressive arthritis group. These observations indicate that the severity of inflammation might be different between the groups when the joint symptoms are considered despite similar levels of rheumatoid factor and c-reactive protein.

Swollen joint counts are routinely used to establish disease activity in rheumatoid arthritis [39]. In Table 5 the percentages of Cold and Heat patients with a positive score on zero to three of the symptoms ‘warm joints’, ‘swollen joints’ or ‘red joints’ are given. Even though high scores on the three symptoms are indicative for the Heat group of RA patients, 40% of the Cold patients also have a positive score on at least one of the three symptoms (frequencies of the scored categories per class are included as supplementary information Figure S2 and the raw symptom score data for ‘swollen joints’, ‘warm joints’ and ‘red joints’ is included as supplementary Table S1). The data indicate that the same number of patients in both groups are in pain and score a similar severity of pain (data not shown). These symptom observations indicate that the Heat RA group has much more joint problems than the Cold RA group. It would be interesting to further study the role these joint symptoms could play in sub-typing RA.

**Table 5:** Warm, swollen & red joint scores.

# Symptoms	Cold*	Heat*
0	60	26
≥1	40	74
≥2	10	74
3	5	63

* Percentages of subjects showing a positive score on a minimum of 0, 1, 2 or 3 of the symptoms.

Our study shows that the important symptoms for the sub-typing are ‘warm feeling’, ‘pain worsens by warmth and movement’, ‘cold feeling’ and ‘aversion to cold’. This is in agreement with a previous study in which a systems diagnosis of 49 patients with rheumatic diseases revealed ‘aversion to cold’, ‘aversion to heat’ and ‘cold feeling’ as highly relevant for the Cold and Heat ranking of the subjects [23]. The symptom ‘aversion to cold’ and coldness in general was found by others as well [12], [40]. Our study and other studies of the biology of Cold and Heat types of RA patients lead to the hypothesis that the currently unclear effectiveness of cold and heat therapy might be improved by targeting it to the right sub-type [41]–[43].

Anti-citrullinated protein antibody (CCP) is extensively studied as a predictor of disease progression in RA [44]. In our multivariate model, CCP levels were higher in RA Heat patients suggesting that there might be a difference in disease progression between the two subtypes. The clinical parameters with the highest positive loadings were MCHC and IgG, while RDW showed the highest negative loading next to CCP. Some relationships between the Cold and Heat subtype and clinical measurements are nonlinear (see supplementary Figure S1 for the transformations of the variables). High as well as low serum levels off MCHC result in a positively transformed value, which is related to the Heat class. The highest levels of IgG are related to the Cold RA type, while lower levels are not so clearly Cold or Heat related. Blood urea nitrogen (BUN) levels increase in plasma with serious kidney damage [45]. Higher or lower levels of BUN are related to the Heat RA group, average levels are related to the Cold RA group. Alanine aminotranferase (ALT) is an important parameter to distinguish Cold and Heat patients, which has been found to be related to insuline resistance and atherosclerosis risk in RA patients [46].

The metabolomics analysis in this study revealed higher urine levels of 11 acylcarnitines in the Heat group of RA patients. The enzyme carnitine palmitoyltransferase I (CTP I), located in the outer mitochondrial membrane, transfers acyl groups from coenzyme A to carnitines allowing the transport of fatty acids into the mitochondria for β-oxidation. Plasma carnitine levels are maintained by a combination of dietary intake, endogenous synthesis by the liver and kidneys and active renal reabsorption. Acylcarnitines can leave the mitochondria, enter the blood stream and can be excreted in urine [47].

Significantly lower total carnitine and acylcarnitine excretion in urine of RA patients compared to healthy controls has been reported, while free carnitine levels in urine and plasma are equal in both groups [48], [49]. Because dietary intake of carnitines was equal in both groups, an impaired carnitine synthesis was suggested which is controlled by skeletal muscle breakdown. Decreased muscle mass and increased muscle turnover are observed in RA patients, which might be explained by a reduced CTP I activity. This was confirmed by a decreased creatinine and 3-methylhistidine excretion [49]. In this study we find that the levels of a range of acylcarnitines are lower in the Cold RA group than in the Heat RA group. These findings suggest that Cold RA patients have less muscle mass and/or a more pronounced muscle breakdown than RA Heat patients. A limitation of this study is that the plasma acylcarnitine levels are not known. Further studies should therefore be performed to validate the hypothesis that Cold and Heat subtypes of RA patients are related to muscle mass or muscle breakdown.

Decreased urine pantothenic acid and acylcarnitine levels have been shown to indicate an increase in beta-oxidation in patients using PPARα agonist drugs [50]. Peroxisome proliferator-activated receptors (PPAR) activate fatty acid and cholesterol catabolism in the liver, activate gluconeogenesis and regulate amino acid metabolism. In addition, PPAR activates CTP I and CTP II activity. We report lower levels of pantothenic acid and acylcarnitine levels in Cold RA patients which might therefore indicate a difference in the activation of PPAR between the groups.

Interestingly, CTP I activity is inhibited by omega-6 fatty acids while an increased ratio of omega-3 versus omega-6 fatty acids consumption stimulates fatty acid oxidation regulated by CTP I activity [51]. A decrease in CTP I activity also seems to play a role in Chronic Fatigue Syndrome patients. In these patients supplementation with carnitine and acylcarnitine was found to decrease fatigue symptoms [52]. These findings suggest that fatigue in rheumatoid arthritis might be related to a decreased CTP I activity and a changed carnitine homeostasis. Fatigue is one of the most important factors in the disease experience of RA patients [53]. Our findings indicate that urine acylcarnitine levels are an important discriminator between two subtypes of RA patients, which might explain differences in the experience of fatigue. These findings also suggest that carnitine and acylcarnitine supplementation might be beneficial for Cold RA patients and less so for Heat RA patients. Further confirmatory studies should be conducted to prove this hypothesis and develop this targeted treatment option.

Significantly lower DHEAS levels have been reported in premenopausal RA patients and are correlated to low morning cortisol levels and high IL-6 levels indicating a suppression of HPA-axis function [54]. Decreased HPA-axis function is associated with a decreased stress response which results in an inadequate response to stress factors. It is hypothesized that this inadequate response can lead to autoimmune and inflammatory disorders [54]. This study shows a higher DHEAS levels in Heat RA patients compared to Cold RA patients suggesting that the Cold RA group has a more suppressed HPA-axis function. A text mining study [55] has shown that diseases related to Cold according to Chinese medicine theory are more related to hormone function disturbances and Heat related diseases to immune function disturbances, which is in agreement with our findings.

This study provides metabolite, symptom and clinical chemistry profiles for two subtypes of rheumatoid arthritis. The profiles indicate a number of biological processes that seem to be regulated differently in the two groups. RA therapy could be optimized for the two groups in various ways. This study suggests differences in the effects of carnitine and acylcarnitine supplementation for the two groups as well as differences in the effects of hormone treatments such as prednison. We think that the better characterization and biological understanding of Cold and Heat RA offered in this study can be used to tailor therapy to each subgroup. Additionally, this study offers a new subtype to include in studies aiming for an improvement of response to treatment. Finally, the mechanism and effect of specific Cold RA and Heat RA treatment options used in Chinese medicine should be studied and might be integrated in standard disease management strategies by using a standardized Cold RA and Heat RA diagnostic profile.

## Supporting Information

Figure S1 **Transformation plots of the clinical chemistry variables.** On the x-axis the categories of the original discretized variables are represented while on the y-axis the optimally scaled quantifications are shown. A negative quantification corresponds to the Cold classification and a positive quantification to the Heat classification.
(JPG)

Figure S2 **Frequencies of scored categories per class.**
(JPG)

Table S1 **Raw data of ‘swollen joints’, ‘warm joints’ and ‘red joints’ scores acquired by the symptom questionnaire.** Patients were asked to give a score between 1 and 7. For the construction of Table 5 the positive scores, scores higher than 1, were counted for the Cold RA and Heat RA group. In Table S1 mean ranks for the Cold RA and Heat RA groups are given for each symptom and the differences between the groups are evaluated with the Mann-Whitney U test. The scoring of the three symptoms is significantly different between the Cold RA and Heat RA group.
(DOC)

Text S1 **Symptoms questionnaire.**
(DOC)
