# Supplementary figures and images for: Characterization of Rheumatoid Arthritis Subtypes Using Symptom Profiles, Clinical Chemistry and Metabolomics Measurements
Source: PLoS One. 2012 Sep 12;7(9):e44331. doi: 10.1371/journal.pone.0044331 (PMC3440441; doi:10.1371/journal.pone.0044331)

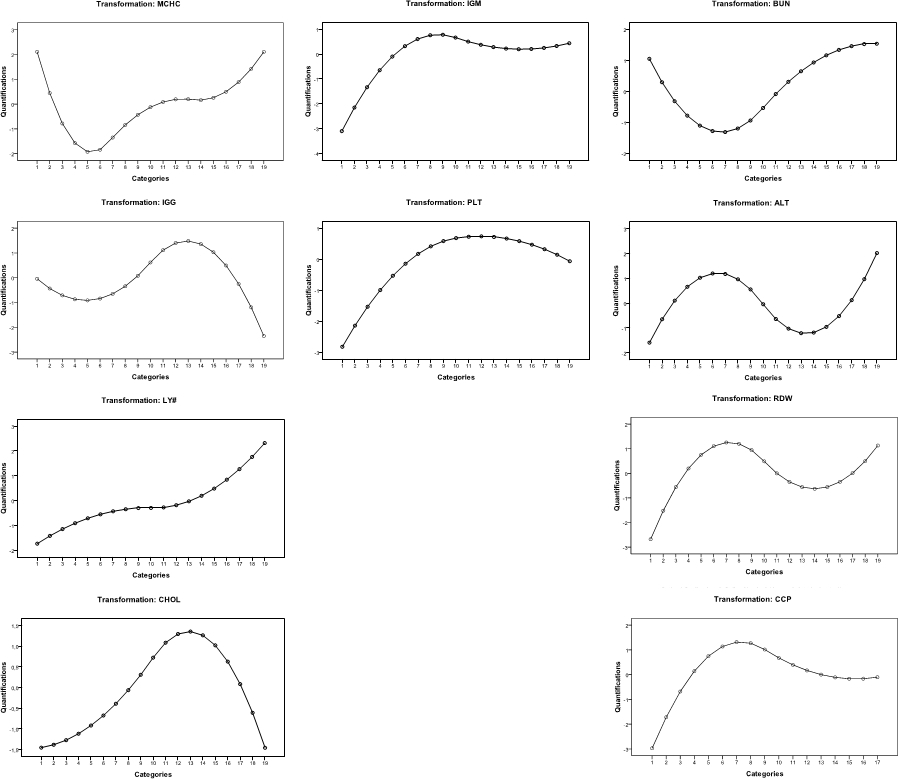

Supplement: Figure S1 — Transformation plots of the clinical chemistry variables. On the x-axis the categories of the original discretized variables are represented while on the y-axis the optimally scaled quantifications are shown. A negative quantification corresponds to the Cold classification and a positive quantification to the Heat classification. (JPG) [file pone.0044331.s001.jpg]

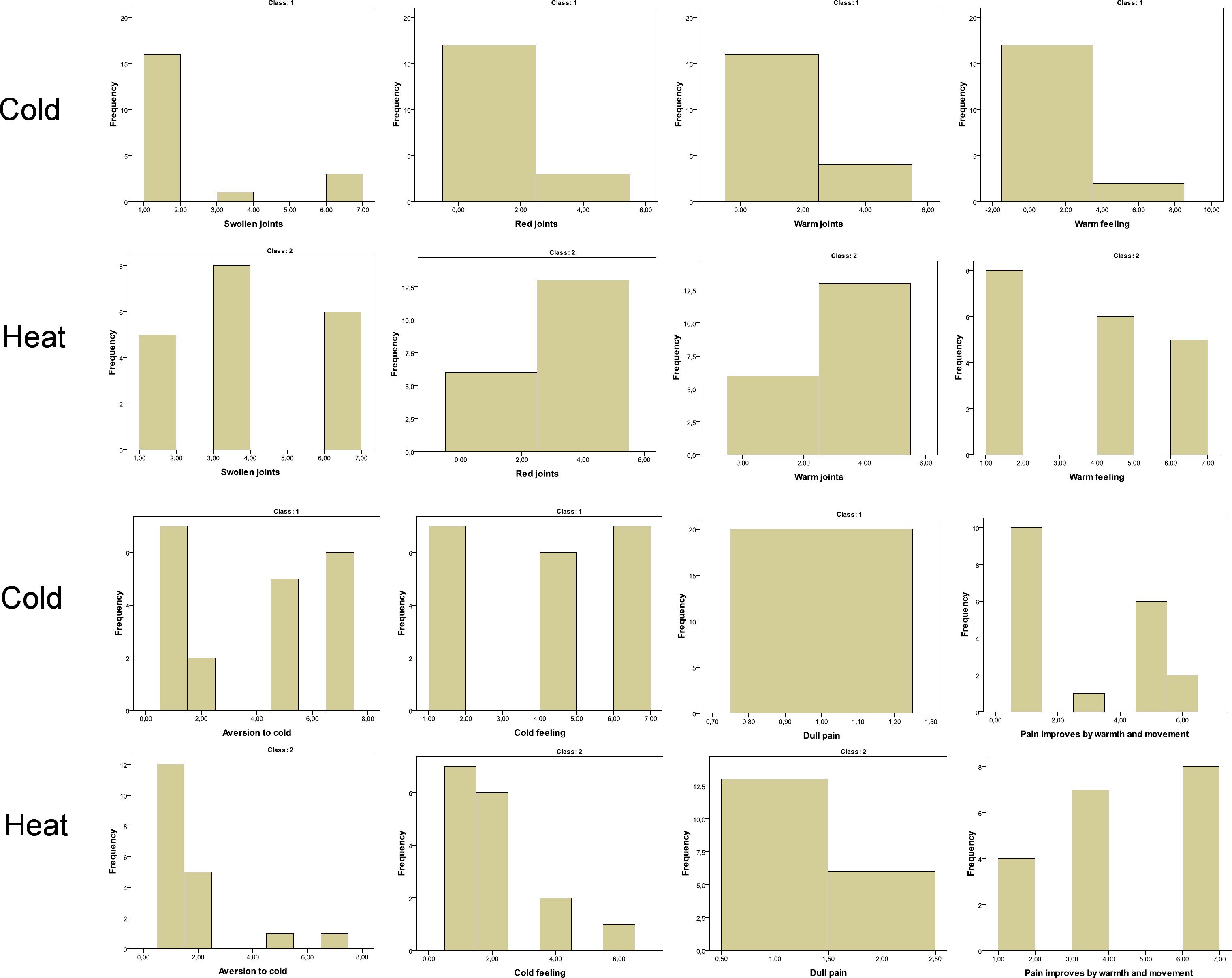

Supplement: Figure S2 — Frequencies of scored categories per class. (JPG) [file pone.0044331.s002.jpg]

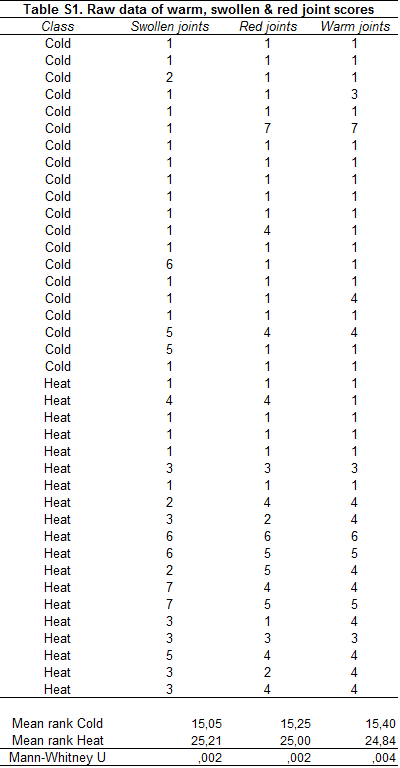

Supplement: Table S1 — Raw data of ‘swollen joints’, ‘warm joints’ and ‘red joints’ scores acquired by the symptom questionnaire. Patients were asked to give a score between 1 and 7. For the construction of Table 5 the positive scores, scores higher than 1, were counted for the Cold RA and Heat RA group. In Table S1 mean ranks for the Cold RA and Heat RA groups are given for each symptom and the differences between the groups are evaluated with the Mann-Whitney U test. The scoring of the three symptoms is significantly different between the Cold RA and Heat RA group. (DOC) [file pone.0044331.s003.doc]
