# Supplementary material for: Characterization of Rheumatoid Arthritis Subtypes Using Symptom Profiles, Clinical Chemistry and Metabolomics Measurements
Source: PLoS One. 2012 Sep 12;7(9):e44331. doi: 10.1371/journal.pone.0044331 (PMC3440441; doi:10.1371/journal.pone.0044331)
Supplement: Text S1 — Symptoms questionnaire. (DOC) [file pone.0044331.s004.doc]

**Questionnaire diagnosis of Rheumatoid Arthritis patients** Patient Nr: ______

This questionnaire is meant to be filled in under the supervision of your caretaker. Please answer all the questions. When an answer is not possible, please note this as well. All information will be handled with care and treated with respect.

The questions often require an estimation of the Severity and Frequency of the symptoms. Select a number between 1 and 7 for this, 1 meaning never or not and 7 meaning very severe or very often.

| **1. Breathing** | | **Answer** | |
| --- | --- | --- | --- |
| Do you experience shortness of breath?  - Does this occur suddenly? | | Severity (1-7):  Frequency (1-7):  yes/no | |
| Do you experience dryness of the mouth? | | Severity (1-7):  Frequency (1-7): | |
| Do you experience dryness of the throat frequently?  - Is this dryness located in the upper throat? | | Frequency (1-7):  yes/no | |
| Do you cough with expectoration of phlegm?  - How much phlegm?  - How sticky is the phlegm?  - How colored is the phlegm? | | Frequency (1-7):  Amount (1-7):  Severity (1-7):  Severity (1-7): | |
|  | |  |  |
| **2. Digestion** | | **Answer** | |
| Does your lower abdomen feel tender or very sensitive?  - Is this feeling aggravated by pressure? | | Severity (1-7):  yes/no | |
| Does your abdomen feel swollen or distended? | | Severity (1-7): | |
| Do you have a feeling of fullness in your abdomen? | | Severity (1-7): | |
| Do you have diarrhea?  - Is the diarrhea smelly? | | Frequency (1-7):  Yes/No: | |
| Do you have loose stools? | | Severity (1-7): | |
|  | |  |  |
| **3. Climate** | | **Answer** | |
| Do you feel cold?  - Is this cold located especially in the feet or lower limbs? | | Severity (1-7):  yes/no | |
| Do you experience thirst? | | Severity (1-7):  Frequency (1-7): | |
| Do you experience chills? | | Severity (1-7):  Frequency (1-7): | |
| Do you feel warm? | | Severity (1-7): | |
| Do you experience fever occasionally?  - Is the fever accompanied by slight chills sometimes? | | Frequency (1-7):  yes/no | |
| Do you dislike heat? | | Severity (1-7): | |
| Do you dislike cold? | | Severity (1-7): | |
| Do you experience spontaneous sweating or sweating at the lightest exertion? | | Severity (1-7):  Frequency (1-7): | |
| Is there a preference for cold drinks? | | Severity (1-7): | |
| Is there a preference for warm drinks? | | Severity (1-7): | |
| Is there a preference for cold food? | | Severity (1-7): | |
| Is there a preference for warm food? | | Severity (1-7): | |
|  | |  |  |
| **4. Quality of the symptoms** | | **Answer** | |
| Do the affected parts of your body feel heavy? | | Severity (1-7): | |
| Does your skin feel numb and cold? | | Severity (1-7): | |
| Do you feel stiffness in your joints? | | Severity (1-7): | |
| Do you have swollen joints? | | Severity (1-7): | |
| Is the joint red? | | Severity (1-7): | |
| Is the joint warm? | | Severity (1-7): | |
| Do you have swellings in your body? | | Severity (1-7): | |
| Do your four limbs feel weak? | | Severity (1-7): | |
| Do your tendons feel weak? | | Severity (1-7): | |
|  | |  |  |
| **5. Pain** | | **Answer** | |
| Do you experience pain? | | Severity (1-7): | |
| What kind of pain do you experience? | soreness, stabbing, sharp, deep, heavy, dull, tingling feeling | | |
| Does the pain aggravate with foggy, humid weather? | | Severity (1-7): | |
| Does the pain aggravate at night? | | Severity (1-7): | |
| Does the pain aggravate by coldness and cold weather? | | Severity (1-7): | |
| Is the pain accompanied with redness and swelling? | | yes/no | |
| Does the pain improve by warmth and movement? | | Severity (1-7): | |

I declare to have filled in this questionnaire truthfully.

| Signature patient: |
| --- |

| Signature caretaker: |
| --- |
